# Desalination Performance Assessment of Scalable, Multi-Stack Ready Shock Electrodialysis Unit Utilizing Anion-Exchange Membranes

**DOI:** 10.3390/membranes10110347

**Published:** 2020-11-17

**Authors:** Jan Čížek, Petr Cvejn, Jaromír Marek, David Tvrzník

**Affiliations:** 1 Faculty of Mechatronics, Informatics and Interdisciplinary Studies, Institute of New Technologies and Applied Informatics, Technical University of Liberec, Studentská 1402/2, 46117 Liberec, Czech Republic; petr.cvejn@tul.cz; 2 Faculty of Science, Humanities and Education, Department of Chemistry, Technical University of Liberec, Studentská 1402/2, 46117 Liberec, Czech Republic; jaromir.marek@tul.cz; 3 MemBrain s.r.o., Pod Vinicí 87, 47127 Stráž pod Ralskem, Czech Republic; david.tvrznik@membrain.cz

**Keywords:** desalination, shock electrodialysis, scale-up, porous medium, electrokinetics

## Abstract

Incumbent electromembrane separation processes, including electrodialysis (ED) and electrodeionization (EDI), provide competitive techniques for desalination, selective separation, and unique solutions for ultra-pure water production. However, most of these common electrochemical systems are limited by concentration polarization and the necessity for multistep raw water pre-treatment. Shock electrodialysis (SED) utilizes overlimiting current to produce fresh, deionized water in a single step process by extending ion depleted zones that propagate through a porous medium as a sharp concentration gradient or a shock wave. So far, SED has been demonstrated on small scale laboratory units using cation-exchange membranes. In this work, we present a scalable and multi-stack ready unit with a large, 5000 mm^2^ membrane active area designed and constructed at the Technical University of Liberec in cooperation with MemBrain s.r.o. and Mega a.s. companies (Czechia). We report more than 99% salt rejection using anion-exchange membranes, depending on a dimensionless parameter that scales the constant applied current by the limiting current. It is shown that these parameters are most probably associated with pore size and porous media chemistry. Further design changes need to be done to the separator, the porous medium, and other functional elements to improve the functionality and energy efficiency.

## 1. Introduction

As the human population keeps growing, the demand for freshwater is predestined to grow as well. With the world population estimated at 10 billion in 2050, the current water scarcity, already present in certain regions, is a problem that needs to be addressed as soon as possible. However, it is not only more people that drive the need for water. The water use has grown at more than twice the rate of the population growth in the last century [1]. Evolving technologies, agriculture, and people’s overall wellbeing go hand in hand with the increased use of the most basic, yet possibly the most precious resource, water. There is also another side of the problem that arises from the management of the used water, which is very often unsuitable for further usage in industry or for normal individual consumption. Of course, there is a finite and constant amount of water on the planet. Therefore, it is our essential yet extremely challenging task to manage water resources sustainably and to treat both the natural resources and wastewaters as efficiently as possible.

In the past decades, there has been great progress made in this cause. Obviously, the development of water treatment technologies has focused on the desalination of sea and brackish waters, which provide the largest, although in the raw state generally not useful, source of water. Membrane technologies quickly became the dominant processes offering high production capacities and feasible operational requirements thanks to intensive research and development in the past years. With a 69% share of the desalinated water produced worldwide in 2019 and the total capacity of 65.5 million m^3^/day, reverse osmosis (RO) is currently the most advantageous technology to operate in large scale desalination plants [2] without any doubt. However, being a pressure-driven process, RO still encounters its limits connected with fouling and scaling, which adds significant maintenance costs. The necessity to overcome the osmotic pressure, which in the case of seawater (with the salinity of approx. 35 g/L TDS), is about 2.3 MPa, significantly drives up the energy requirements and/or lowers the water recovery of the systems [3]. On top of that, RO removes practically every non-water substance from the feed stream, which makes it unsuitable for selective decontamination [4].

Electrodialysis (ED), which has been commercially exploited for 70 years now, is another water treatment method, using selective transport of ionic species in an electric field across ion-exchange membranes [5]. This process offers high energy efficiency for desalination in the range of concentrations between ca. 500 and 5000 ppm and is, therefore, more suitable for low-salinity brackish waters [6,7]. Thanks to being able to selectively separate charged species from non-ionic ones, ED finds use in the treatment of industrial wastewaters, including removal and recycling of heavy metals (nickel) from rinse waters, inorganic acid regeneration in the chemical industry, reacidification of fruit juices, desalination of whey in the food industry, and more [8,9] On the other hand, the effectivity of ED falls significantly as the feed stream becomes more diluted (<100 ppm). The high ohmic resistivity drives the energy consumption up. Therefore, ED cannot be used for complete water purification purposes. In electrodeionization (EDI), a related method, this is solved by filling the dilute chambers with ion-exchange beads. Ion-exchangers are characterized by relatively high conductivity and provide a large active ion-exchange area. As a result, it is possible to obtain a concentration of ionic and ionizable species far below ppb levels. Therefore, EDI is a method that is used in the electronic, pharmaceutical, and chemical industries, where ultra-pure water with conductivity lower than 0.1 μs/cm is needed.

Although further development of these currently quite well-understood processes continues, there is only so much that can be done to optimize the components and materials used for certain applications before reaching the physical limitations each of the current technologies entails. The other way to look at the research for new and more effective methods is to revise the fundamentals of the current technologies using state-of-the-art tools that modern science provides. Shock electrodialysis (SED) is one of the rising technologies to be developed using this approach by overcoming the limits of classical ED and practically using them to one’s advantage. SED utilizes ion concentration polarization (ICP) to produce fresh, ion-free water by deionization shocks in porous microstructures, which extend the boundary layers adjacent to the ion-exchange element in the overlimiting current (OLC) region.

### Development and Theory of Shock Electrodialysis

**Figure 1:**
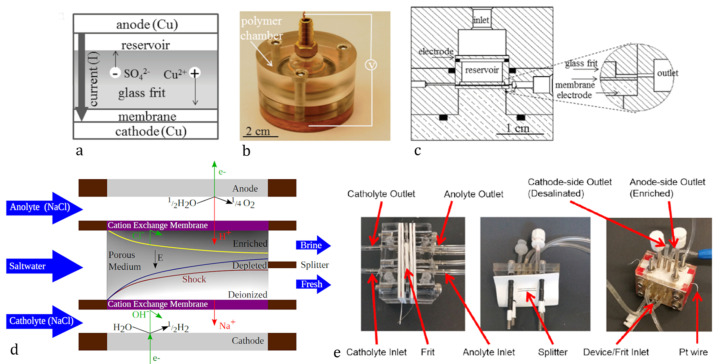
Shock electrodialysis device development. (**a**) shows the scheme of the “button” unit displayed on (**b,c**) [17]. (**d**) shows the operating principle of the “second generation” unit that is displayed in (**e**) and has overall dimensions “2 × 2 × 1.5” [20]. Deionization shocks spread through the channels of the porous medium as a sharp concentration gradient. From these extended zones, freshwater and brine are separately collected. The outlet points are separated by a splitter, a thin partition that prevents the outlet streams from mixing once discharged from the device [20]. Adapted from [17] for (**a**,**b**,**c**) and [20] for (**d**,**e**). Copyright 2013 and 2015. American Chemical Society.

Deionization shocks were first described and experimentally confirmed on nano-microfluidic lab-on-a-chip devices by [10,11]. At the interface of a negatively charged microchannel and a nano-channel filled with electrolyte, a propagation of ICP from the ion-exchange element (nano-channel) in both directions was observed. Forming a sharp concentration gradient, this manifested itself as a deionization shock. To utilize this mechanism at a larger scale, Mani and Bazant [12] extended the models to complex microstructures with many interconnected micro- and nano-channels, where the overlimiting current to propagate the deionization shocks was predominantly driven by the surface conduction (SC) in smaller (around 1 μm) and electroosmotic flow (EOF) in larger (around 100 μm) channels, as) described in [13]. A device for water purification based on these principles was proposed in patents by Bazant’s group at the Massachusetts Institute of Technology (MIT) [14,15]. The term Shock electrodialysis was first used in the work of Deng et al., where a laboratory-scale water purification device was used to demonstrate the capabilities of shock deionization using a negatively charged silica glass frit as a porous microstructure that was sitting on a cation-exchange membrane, as shown in Figure 1 [16,17]. Further works were published between 2015 and 2020 by prof. Bazant’s group demonstrating the functionalities of improved and scale friendly designs of a new shock electrodialysis unit that once again utilized a porous frit with negative surface charge sandwiched between a pair of cation-exchange membranes to induce the concentration polarization. The device was capable of reducing the ion concentration by four orders of magnitude [4,18,19,20].

**Figure 2:**
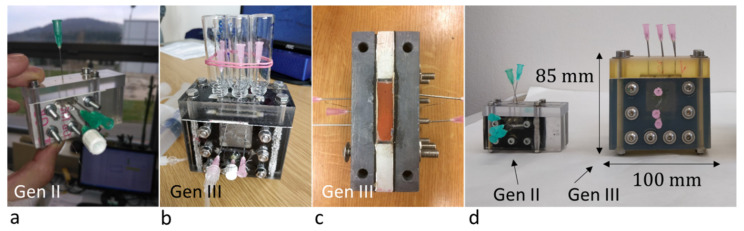
Comparison of “Gen. II” and “Gen. III” units built at TUL. (**a**) Gen II was built based on [21] (**b**) Gen III unit made of transparent polycarbonate, (**c**) top-down view of Gen. III unit made of polyvinyl chloride, without the separator. (**d**) comparison of the latest (3D printed) Gen. III unit with the Gen. II unit.

In a sense, the SED unit design is rather similar to that of a classical electrodialysis module. However, as the principle is based on the extension of ion depleted (and enriched) zones in the porous medium, the concentrate and diluate are collected from the same single “desalination chamber”. To promote the desired effects associated with OLC, the thickness of the porous material (and therefore the chamber) is limited. That limits the amount of water produced and brings difficulties with product collection at such a tiny scale. The early experiments of our group at the Technical University of Liberec (TUL) focused on the first scale-up of the unit (what we call “Generation III unit”) inspired by the Bazant Research Group’s work (referenced to as “Generation II unit”) (Figure 2). The unit was made twice as large in all dimensions with an active membrane surface eight times larger (a surface in contact with the porous medium) compared to the unit (“Gen. II unit”) presented in [20]. That allowed for approximately ten times higher flow rates through the porous medium (Figure 2). We also experimented with various types of porous materials. Interestingly, very simple and not well-defined materials, such as simple fired brick, delivered performance similar to the glass frit [21]. However, the desalination performance of the unit was not on par with the results published by Bazant’s group, and we still experienced difficulties with product collection [22,23]. Table 1 compares the results achieved on our “Gen. II” and “Gen. III” units to the results published by [20] using “dimensionless current” Equation (1) proposed in his work (and the work of [17], that collapses the desalination data on a single desalination curve, where desalination is a function of dimensionless current Ĩ described as

(1) $$\tilde{I}=\frac{I}{zcFQ}$$

where *I* is the applied current, *z* is the cation charge, *c* is the cation concentration, *F* is Faraday’s constant, and *Q* is the volumetric flow rate of the feed. When using a solution with only single species of cation present and assuming the anion transport across the cation-exchange membrane is zero, the denominator in Equation (1) presents diffusion-limited current defined as the rate of forced convection of positive charge carriers [18]:

(2) $$I_{\lim}=\sum_{i}z_{i}c_{i}FQ$$

where the *i* index corresponds to a specific cation present in the solution. When combining positively charged porous material with anion-exchange membranes, the current is limited by anions, whereas it is assumed that there is no transport of cations across the anion-exchange membranes.

In this work, we present a new, larger SED unit with optimized components and a multi-stack friendly design inspired by the previous work and experience. Furthermore, for the first time in SED development, we have utilized anion-exchange membranes to separate anions. We report data obtained from basic experiments showing single salt electrolyte desalination performance in a galvanostatic regime. We once again compare the data to the previous generation units, assess the energy consumption of the device, and discuss the possible improvements in design for future work.

## 2. Materials and Methods

**Figure 3:**
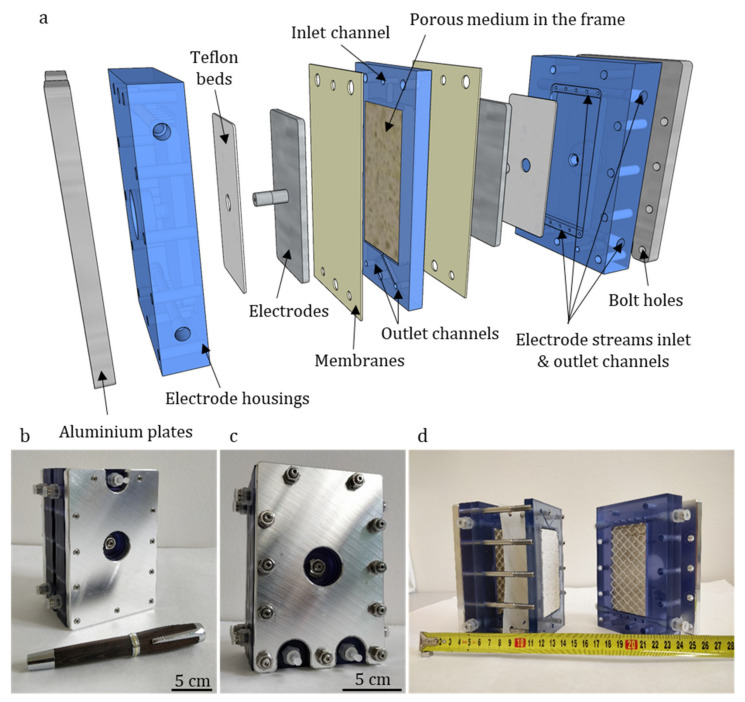
(**a**) Disassembled model of “Generation IV” unit showing all its components, (**b**) inlet side of the unit, (**c**) outlet side of the unit, (**d**) disassembled unit and measuring tape in centimeters.

The device as shown in Figure 3 was designed and fabricated in cooperation with MemBrain s.r.o. (Stráž pod Ralskem, Czech Republic) and Mega a.s. (Stráž pod Ralskem, Czech Republic) companies.

The unit casing was composed of five separate parts-two structurally rigid aluminum plates (150 × 100 × 10 mm) on the outside, two housings for the electrodes (150 × 100 × 25 mm), and a porous medium frame in the middle (outer dimensions 150 × 100 × 10 mm). Both the electrode housings and the porous medium frame were 3D printed using photopolymer. The whole unit was screwed together by eleven 6 mm diameter bolts.

**Figure 4:**
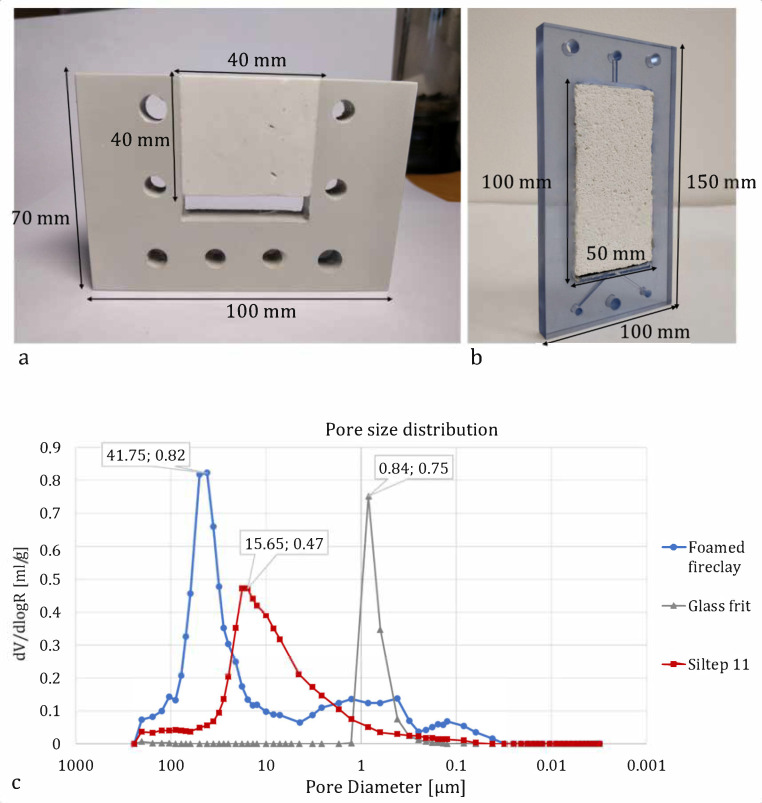
Comparison of (**a**) frame with a porous medium installed for “Gen. III” unit and “Gen. IV” unit. In (**a**), the separator is not included but sits on top of the exposed side of the porous medium instead. In (**b**), the separator is integrated down at the bottom of the frame. The thickness of the parts is 10 mm both in (**a**,**b**). (**c**) shows frequency curves for the materials used and glass frit used before as a reference.

The porous medium frame and the splitter were significantly redesigned compared to the previous unit. Instead of a U-shaped frame, where the porous medium was glued on two sides, leaving space for the reservoir at one side and the other one open to connect the splitter as a separate part, the current solution used a 100 × 50 × 10 mm large frame that was closed all around (Figure 4a,b). The solution was fed into the material through an inlet channel with a small extra space left at its opening to distribute the solution across the top area of the medium, where it enters. The splitter was a 3 mm thick, rigid partition integrated into the bottom of the frame midway along the edge, leaving equal space at each side for the outlets. Streams from both sides of the splitter are then collected each by means of a single channel to the outlet points.

The materials for the porous medium were chosen based on a previous positive experience with refractory ceramics, mainly for its chemical and mechanical stability [21]. The porous medium was shaped into 100 × 50 × 10 mm blocks of refractory ceramics Siltep 11 and foamed fireclay material (supplied by Silike keramika s.r.o., Děčín, Czech Republic). Siltep 11 is composed of 55% SiO_2_ and 35% Al_2_O_3_ (the rest being other inorganic oxides and organic filler spheres). The foamed fireclay consists of 68% Al_2_O_3_ and 28% SiO_2_, the rest being other inorganic oxides. Typical pore diameters of 15.6 μm and 41.8 m and porosity of 51% and 63% for Siltep 11 and foamed fireclay respectively were estimated using mercury porosimetry (with the help of Unipetrol Centre for Research and Education, Ústí nad Labem, Czech Republic) (Figure 4c)). Once fitted into the frame and sealed at the interfaces using silicone sealant, a medium was again sandwiched between a pair of ion-exchange Ralex^®^ AMPES (anion-exchange, MEGA a.s., Stráž pod Ralskem, Czech Republic) membranes so that contact between the medium and the membranes was ensured. These membranes, manufactured by Mega a.s. in the necessary shape and size to fully cover the frame and outer casing parts, were chosen for experiments reported in this paper as well characterized, commercially available, and reliable membranes. In other experiments we performed, ion-exchange membranes developed at TUL were also successfully utilized, delivering similar performance [24]. Outer casing parts house the 100 × 50 × 5 mm platinum-coated electrodes leaving enough space for the circulation of catholyte and anolyte, which were fed and collected into the chambers through holes drilled at the top and bottom of the chamber. The electrode chambers were filled with plastic mesh to keep the membranes adjacent to the porous medium and to avoid detaching. The electrodes were supported using soft teflon (PTFE) gasketing and led out through the casing to be connected to the ZHAOXIN KXN-2005D (purchased from Tipa, spol. s.r.o., Opava, Czech Republic) stabilized DC source using 0.25 mm dia titanium wires. The unit was operated galvanostatically to sustain a stable overlimiting current. The applied current was calculated using an equation derived from a formula for dimensionless current [Equation (1)] to set a series of variable dimensionless currents as the parameter to estimate the performance of the device and compare it to the results published by Bazant’s group at MIT.

**Figure 5:**
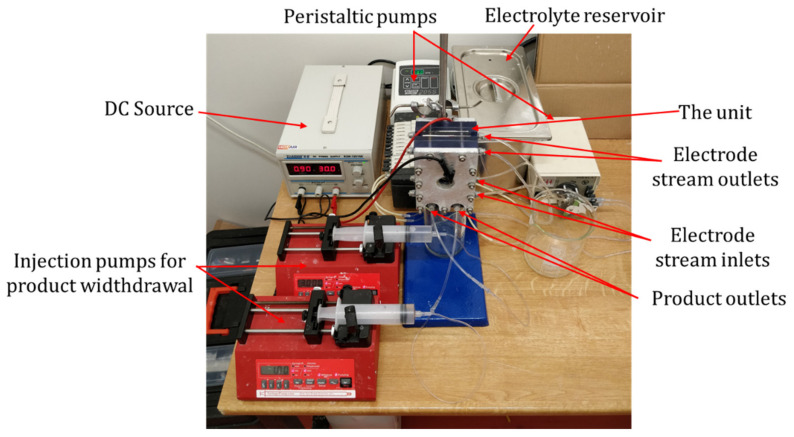
Testing setup. In this experiment, products were withdrawn using injection pumps, which is a method not employed to obtain the presented data. Otherwise, the setup is identical to the used one. The picture serves an illustrative purpose only.

The solutions were prepared by dissolving 1.42 ± 0.01 g Na_2_SO_4_ or 0.59 ± 0.01 g NaCl per every liter of deionized water to obtain 10 mM electrolyte. The solution was pumped into the unit using Kouřil Co. PCD 61.4 peristaltic pumps (Kyjov, Czech Republic) for inlet into the porous medium and Watson Marlow 205S (purchased from AxFlow s.r.o., Prague, Czech Republic) ones for transporting electrode solutions through polyurethane tubing connected to the unit via push-in fittings as three separate streams. The flow rates were set to 2.4 or 4.8 mL/min for feed and 7.4, 9.6, or 17.2 mL/min for anolyte and catholyte separately. To ensure the electrode chambers were filled with electrolyte completely, catholyte and anolyte were fed into the unit through the bottom and discharged through the top of the electrode chambers. The feed was pumped into the unit from the top, and products were collected at the bottom. The experimental apparatus is shown in Figure 5.

Samples were collected as they were spontaneously discharged from the unit into beakers. The unit was stored in demineralized water between experiments. Therefore, at the beginning of each experiment, the electrolyte was passed through the unit for approximately 20 min to ensure the porous medium, and the electrode chambers were filled with electrolyte before connecting the unit to the DC source. Once connected, the voltage was allowed to stabilize, if possible (in some experiments, the voltage did not stabilize, as discussed in *Results* and *Discussion*). Products, as well as electrode outlet streams (mixed together), were characterized by measuring volumes, conductivity using WTW Cond 3310 together with WTW TetraCon 25 cell, and pH using WTW pH 3310 with a SenTix^®^41 cell, all purchased from WTW (Prague, Czech Republic).

Deionization performance D (hereinafter referred to as ion-removal) was estimated based on conductivity measurements as

(3) $$D=\;\frac{1-\kappa }{\kappa_{0}}\;\times 100\;\%$$

where κ is the conductivity of the diluted stream and κ_0_ is the conductivity of the feed. This method had been chosen due to the lack of equipment in our laboratory. More precise estimation could be obtained by measuring concentrations using spectroscopic methods because conductivity might be influenced by the H^+^ and OH^−^, products of present water splitting [19]. However, as we compared the ratio of product conductivity to feed conductivity, the obtained deionization factor was considered illustrative enough to count with.

## 3. Results

### 3.1. Desalination Performance

**Figure 6:**
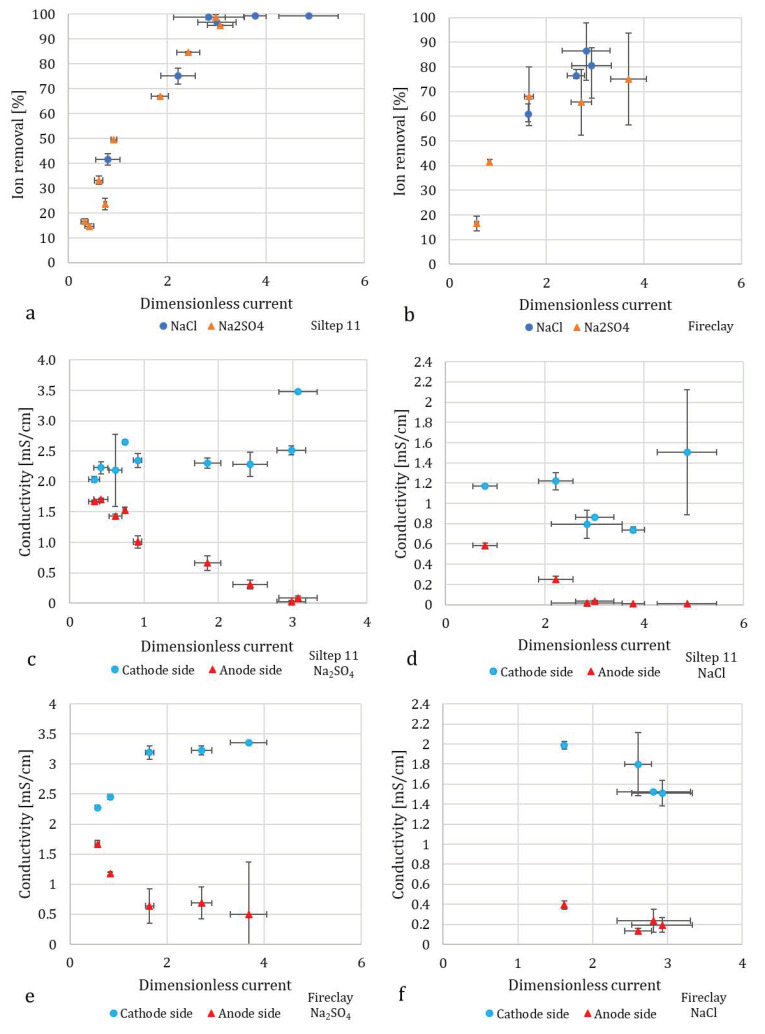
Experimental data for the galvanostatically operated unit with Siltep 11 and foamed fireclay as the porous medium, AEMs, feed concentration 10 mM. (**a**,**b**) show average desalination as the function of dimensionless current, (**c**–**f**) show the conductivity of cathode and anode side during these experiments for all the combinations of porous materials and electrolytes. The rather big deviations suggest unstable performance in some cases, which could have been caused by unstable flow rates (visible in (**a**,**b**) as error bars for the dimensionless current) and pH changes as discussed.

**Figure 7:**
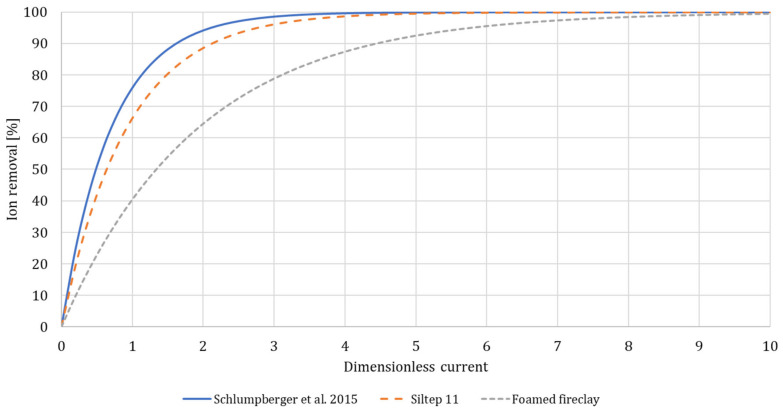
Comparison of performance of our unit with various installed porous media and AMEs and reference units with silica glass frit and cation-exchange membranes (CEMs) using the model (4) to fit the data.

As expected, an increase in dimensionless current led to increased desalination, as displayed in Figure 6a,b. For both NaCl and Na_2_SO_4_ with Siltep 11, the desalination reached 99% around a dimensionless current of three. For foamed fireclay, the desalination did not even reach 90% for a dimensionless current of three (and more for Na_2_SO_4_). We compared these data using a model reported by [20], who observed quite a precise collapse of all the measured desalination data on a single master curve approximately following exponential equation

(4) $$\log\frac{c}{c_{0}}=\alpha \tilde{I}$$

where parameter α= −0.619. For our case, this parameter, α=−0.472±0.031, for Siltep 11 and α= −0.220±0.020 for foamed fireclay. Finally, plotting the master curve for each porous material and Schlumpberger’s data respectively provided a good comparison of the desalination performance with dimensionless current as the single variable parameter (Figure 7). We could clearly see both of our cases fell behind the unit with glass frit, with foamed fireclay reaching the desired desalination at a very high dimensionless current of approximately ten and more. In practice, reaching such a large dimensionless current would require applying a current of 0.48 A for Na_2_SO_4_ and 0.24 A for NaCl (with 10mM solution and flow rate 1.5 mL/min), which would drive the voltage well above 200 V with our unit. There are a few possible reasons for the worse performance of foamed fireclay. At first, it could be that the pore size was too large to sustain uninterrupted deionization shocks along the channels (as explained above in the Section 3.4: *Porous Media Choice and Shape*). Second, it might have been the surface charge and pH changes, respectively. The fireclay used is a material composed mostly of alumina and silica, with alumina being prevalent (2.4:1 Al_2_O_3_:SiO_2_). The average pH of the diluted stream (anode side) for experiments with Na_2_SO_4_ was 9.9 ± 0.4, indicating a negative charge of both silica and alumina (IEP = 3.9 and 8.8 respectively [25], and 6.8 ± 2.4 for NaCl, indicating more-less positive charge for alumina (and negative for silica), forming the overall rather positive charge, although probably reduced by the negative silica. With Na_2_SO_4_ and anion-exchange membranes (AEMs) used, the negative charge would not (in theory) promote deionization shocks in the desired way because the anions would be driven along the charged walls in the opposite direction. That said, zeta potential as a function of pH of the material should be measured to verify this hypothesis. The observed performance suggests only limited deionization shock propagation and functionality. For Siltep 11, the pH varied significantly between 3 and 11 for Na_2_SO_4_ and 6.5–9 for NaCl without any correlation with the applied current or desalination performance. The cause of these significant, rather random changes in pH, is unknown.

It is important to note that anion-exchange membranes were used in these experiments, whereas we compared the results to a unit with cation-exchange membranes. The combination of positively charged porous material and AEMs should promote deionization shocks to effectively separate the anions from the bulk solution. This explains the somewhat lower desalination performance observed with Na_2_SO_4_ compared to NaCl, as the diffusivity coefficient of sulfate anion is approximately twice lower than that of Cl^−^ (≈ 1.07 × 10^−9^ m^2^/s for SO_4_^2−^ compared to 2.03 × 10^−9^ m^2^/s for Cl^−^ [26]), which leads to lower ion mobility despite the higher negative charge.

### 3.2. Flow Rates and Water Recovery

As far as water recovery is concerned, the obtained data is again in conflict with the observations reported by [4,18,19,20], as described above. The water recovery stayed rather the same, moving in the range of 45% to 55%, occasionally dropping below 40% or rising above 60% without any apparent reason. That said, the splitter was positioned in the middle along the outlet edge, and therefore water recovery of 50% would be expected without the effects of EOF. The changes in values were probably connected to the unstable flow rates through the porous medium that was present despite the fixed flow rate of the inlet. The flow rate Q used in Equation (1) to estimate the dimensionless current was, therefore, the actual flow rate measured at the outlets. The inconsistency may have added errors to the experiments, while the reason behind this behaviour is not clear. One possible explanation is the change of hydrodynamic resistivity of the porous material caused by some sort of impurities from the feeding solution or, more likely, debris and loose particles released from the porous material itself, which may have blocked channels at the output. To minimize the influences caused by the medium, the porous material needs to be revised. Structural irregularities present in not very well-defined material, which the Siltep 11 mainly is, may have interfered with the flow rates and water recovery as well. Besides these effects, peristaltic pumps are not ideal for driving the solution into the unit because of the pulsating flow produced by these systems. To minimize these effects, either pulsation dampeners or flow buffer capacitors developed later by Bazant’s group could be used to smooth out the pulses and stabilize the flow rates [19]. In future work, we are planning to test product withdrawal using peristaltic or injection pumps, which, based on the initial experiments we have performed, seemed to work rather well, even stabilizing the desalination.

### 3.3. Energy Consumption

**Figure 8:**
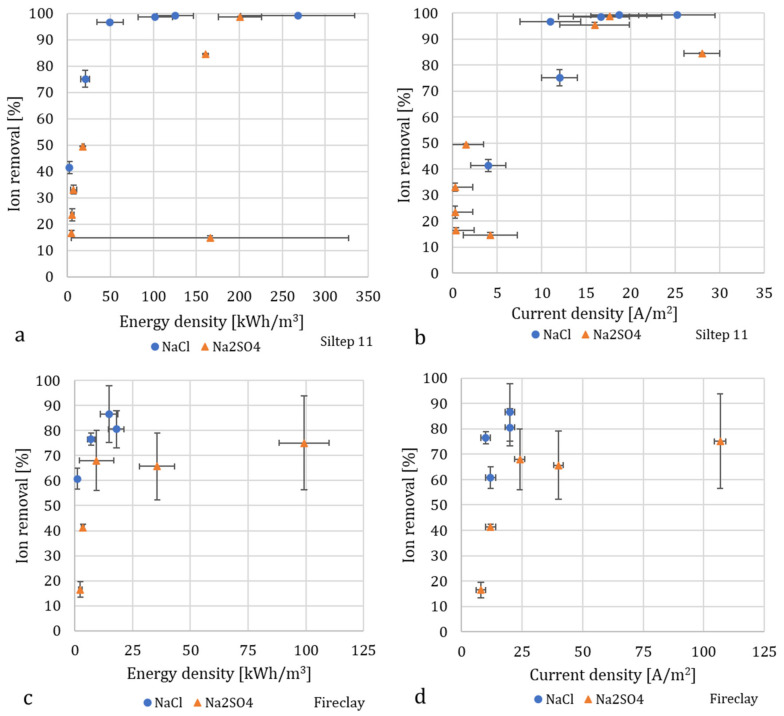
Desalination as a function of energy density and current density for Siltep 11 in (**a**,**b**), foamed fireclay in (**c**,**d**), and NaCl and Na_2_SO_4_ as electrolytes. The data show inconsistencies, for instance, the high energy density and low desalination for one case in (**a**), which was caused by the high voltage that was necessary to sustain the applied current. This may have been caused by temporary scaling inside the porous material or on membranes. The energy required for pumping is not included.

Figure 8a–d shows the energy consumption and current density required to achieve a certain percentage of desalination as calculated from the performed experiments (considering only the electrical component and not the pumping). The energy density needed to reach 90% (or close to 90% in the case of fireclay) desalination was notably lower for NaCl with both Siltep 11 and foamed fireclay, as expected due to the size and charge of SO_4_^2−^. After reaching desalination of 99%, the energy requirements for additional desalination rise steeply. Further increments in applied current and voltage also caused heating of the unit, mainly apparent in the electrode streams, in which the temperature increased up to 40 °C while the unit got warm to the touch. Lowering the energy demand for production of very diluted (>99%) water could be possible by splitting the process into multiple stages in series, a method that is used in commonly employed desalination technologies and was tested for the first time with SED by [18]. Despite the suboptimal choice of porous material (especially regarding the combination of surface charge and choice of membranes) and the energy losses due to heat generation, the energy consumption is comparable to experiments performed on glass frit and in much smaller volumes by [20]. This observation is interesting from the scale-up point of view. The larger cross-sectional area of the frit used in our unit decreases energy consumption as, according to Ohm’s law, the power *P* is inversely proportional to the cross-sectional area (*P* = I^2^R, where resistivity R ~ L/A; L being the length of the resistive material and A the cross-sectional area). Comparison between the L/A ratio of the porous material of our unit (L/A = 10/5000 mm^−1^ = 0.002 mm^−1^) and the glass frit in early SED unit by [20] (L/A = 2.7/200 mm^−1^ = 0.0135 mm^−1^), unveils the reason behind similar energy density even at two orders of magnitude higher flow rates, which drives the energy requirements up (the current I is proportional to flow rate Q for the same dimensionless current according to Equation (1) and, therefore, E = P/Q ~ QL/A [18]). Although the power consumption was still significant, this study was not primarily focused on its reduction, and the main components to affect it remain largely unoptimized.

### 3.4. Porous Media Choice and Shape

The theoretical and experimental work published previously suggests the usage of porous materials with close to 1 μm pore size to sustain deionization shocks in the OLC region [4,13,16,20]. Larger pores (approximately >10 μm) may lead to convective mixing that may interrupt the shock formation and produce instabilities inside the porous material. However, such a small pore size (around 1 μm) is inevitably connected with high hydrodynamic resistivity. Therefore, low volume flow rates or increasing energy demand for pumping seem to be the downsides of the previously described SED devices [21]. As the purpose of this scale-up was to increase the production of freshwater, it was necessary to consider the usage of materials with higher solution throughput, possibly even with lower effectivity in the beginning. Therefore, two types of porous ceramics with different pore sizes that were both more than one order of magnitude larger compared to pores of the glass frit used in [4,16,17,18,19,20] (and different enough one from the other) were chosen for this study. The solution throughput was also related to the increased dimensions of the porous material, especially in the direction of the solution flow, which only added to the hydrodynamic resistivity of the material. On the other hand, this increase was made with an idea of a longer flow trajectory that would provide more space and therefore require lower currents for the shock to propagate, as also [18] suggests and discusses. For further scale-up, widening the porous medium (and the whole unit) could also be beneficial in order to increase the cross-sectional area and the functional membrane area. The size, shape, and morphology significantly affected power consumption.

The key factor of a porous material is its surface charge, which needs to be paired with the right choice of ion-exchange membrane type (anion- and cation-exchange, respectively). It should be noted that neither of the materials selected for this study was probably ideal in terms of chemical composition. Because of the heterogeneity of the materials, it is difficult to estimate the isoelectric point without proper characterization of zeta potentials, and therefore to estimate the surface charge at the microchannels. Unfortunately, such characterization of a bulk porous material requires special cells for electrokinetic analysis or alternative methods that were unavailable to us at the time of research presented in this paper. Nevertheless, the fact that the materials were most probably able to promote anion concentration shocks in the present pH conditions suggests that a wide variety of similar materials could be used. To study the influences of the pH on the electrolyte-porous media interactions, also buffer solutions could be potentially utilized to suppress the pH changes. From what we know so far, a proper choice of material from the chemical perspective may increase the effectiveness of the process and thus lower its energy demand.

### 3.5. The Practicality of Design and Scalability

The ease of workflow constitutes a great benefit of this new and larger design. Previously, tiny components made the unit difficult to assemble, seal, and service afterward. Such difficulties have been significantly reduced. Besides the obvious goal to increase the capacity of the device from the performance point of view, the larger throughput of the device also allows for more samples to be collected in shorter periods of time, which speeds up the process of testing.

Changes to the separator were described above in the Section 2: *Materials and Methods*. The experiments have proven this design to be functional both in terms of separation and integration into the device itself. Being in between the membranes that cover the whole area, the sealing of the unit is no longer that difficult, and technically, one less part (the separator in previous generations) is needed. An important feature of the frame and separator being a single component is that it allows the unit to be easily scaled-up into multi-stack arrangement only by adding more frames with functional porous media, which will be executed in our future work.

The potential drawbacks of this design include the impossibility to reposition the separator as it is integrated into the middle part. Therefore, the whole unit needs to be disassembled in order to change the frame for the porous material. Also, the separator in our testing unit is thick (3 mm), which may still cause mixing as the solution is driven towards it or affect the water recovery. Other future design changes would include revising the plastic material for the casing and the system of bolts that hold the parts together. During our experiments at higher voltages, the unit got warm to the touch (as discussed in Section 3.3: *Energy Consumption*), which, in combination with pressure presented by the screws, deformed the casing and the media frame and made it very difficult to disassemble and reassemble again while keeping it sealed.

## 4. Conclusions

Scalable design is an essential criterion for every new technology to be useful in practice. Up until now, shock electrodialysis was demonstrated only on very small-scale laboratory units. We built and demonstrated functionalities of a larger unit with 10–100× larger capacity while using two different porous materials with one order of magnitude larger pores. Results showed more than 99% ion removal in a one-step desalination process of single electrolyte solutions without any significant energy consumption increments, compared to previously tested designs due to the size and shape changes to the medium. With a few design changes, this unit would be ready to be rebuilt into a multi-stack device and possibly scaled into larger dimensions to significantly decrease the energy requirements, both of which are the goals of our future work. While SED cannot compete with common desalination technologies yet, cheap components and materials, together with its attractive functionalities, may offer an interesting solution for small-scale operation in freshwater production and selective water treatment in the future.

We have shown that the shock electrodialysis and deionization shock propagation is not limited only to cations and cation-exchange membranes, respectively, but can work with anion-exchange membranes as well, while obtaining a high degree of desalination. The results confirm the suitability of scaled (dimensionless) current as a single variable parameter to estimate and compare the performance of the device in the overlimiting region, as long as the porous medium is well characterized. Besides the shape and size, it is the pore size that affects the desalination performance. We have shown that material with pore sizes of around 40 μm most probably decreases the desalination performance significantly, while pore sizes around 16 μm provide a relatively good balance between a good solution throughput and deionization shock propagation. That said, these results need to be verified once the surface charge of both materials is characterized and compared. The desalination performance, stability, and possibly water recovery should also be further improved by also securing constant flow-rates and adjusting the thickness and position of the separator.

## Figures and Tables

**Table 1:** Comparison of desalination performance between our units built at the Technical University of Liberec (TUL) and the results reported by [20] (marked as Reference units) at the same dimensionless current, a parameter defined by Equation (1). The number presented in the reference unit column expresses the desalination performance of Schlumpberger’s reference unit at the same dimensionless current as was used to characterize the Gen. II and III units, respectively. The “Gen. II unit” used silica glass frit and a design very similar to the reference unit, and the “Gen. III unit” was a scaled-up unit described above, fitted with silica ceramics with the porosity and pore size comparable to the glass frit.

	Desalination (%)		
Dimensionless Current (1.1.)	Gen. II Unit	Gen III Unit	Reference Unit
Ĩ ≈ 0.4	36	-	≈ 40
Ĩ ≈ 0.6	-	40	≈ 60
